# Interference competition pressure predicts the number of avian predators that shifted their timing of activity

**DOI:** 10.1098/rspb.2018.0744

**Published:** 2018-06-06

**Authors:** Yifan Pei, Mihai Valcu, Bart Kempenaers

**Affiliations:** Department of Behavioural Ecology and Evolutionary Genetics, Max Planck Institute for Ornithology, Eberhard-Gwinner-Str. 7, 82319 Seewiesen, Germany

**Keywords:** time partitioning, interference competition, species richness, Accipitriformes, Strigiformes, body size

## Abstract

Being active at different times facilitates the coexistence of functionally similar species. Hence, time partitioning might be induced by competition. However, the relative importance of direct interference and indirect exploitation competition on time partitioning remains unclear. The aim of this study was to investigate the relative importance of these two forms of competition on the occurrence of time-shifting among avian predator species. As a measure of interference competition pressure, we used the species richness of day-active avian predator species or of night-active avian predator species (i.e. species of Accipitriformes, Falconiformes and Strigiformes) in a particular geographical area (assemblage). As an estimate of exploitation competition pressure, we used the total species richness of avian predators in each assemblage. Estimates of the intensity of interference competition robustly predicted the number of Accipitriformes species that became crepuscular and the number of Strigiformes species that became day-active or strictly crepuscular. Interference competition pressure may depend on body size and on the total duration of the typical active period (day or night length). Our results support—to some extent—that smaller species are more likely to become time-shifters. Day length did not have an effect on the number of time-shifter species in the Accipitriformes. Among the large Strigiformes, more time-shifter species occur in areas where nights are shorter (i.e. where less of the typical time resource is available). However, in the small Strigiformes, we found the opposite, counterintuitive effect: more time-shifters where nights are longer. Exploitation competition may have had an additional positive effect on the number of time-shifters, but only in Accipitriformes, and the effect was not as robust. Our results thus support the interference competition hypothesis, suggesting that animals may have shifted their time of activity, despite phylogenetic constraints on the ability to do so, to reduce the costs of direct interactions. Our findings also highlight the influence of body size as a surrogate of competitive ability during encounters on time partitioning, at least among avian predators.

## 1. Introduction

Species vary widely in their timing of activity. Niche theory considers time as a resource and suggests that the timing of activity is a plastic trait driven by the intensity of interspecific competition [1–5]. Some species have become active outside of the time period typical for the taxonomic order to which they belong and are referred to as ‘time-shifters’ or ‘time-shifted species'. Previous studies have linked the evolution of nocturnality and the existence of time-shifters to two forms of competition [6–8]: (i) direct interference competition (i.e. competition by defending resources and imposing harm on competitors, which is size-dependent) and (ii) indirect exploitation competition (i.e. competition among sympatric species by resource depletion without direct contact, independent of size or competitive ability).

Direct interference competition can be avoided if the active times of sympatric species do not overlap [3–5]. Time is then seen as an orthogonal niche dimension [4] that is independently partitioned among competitors. When animals are active at different times, they can also avoid or reduce exploitation competition if they then also use different food or other resources [2]. In the latter case, the partitioning of time is not independent of the partitioning of these other resources. Such a scenario is likely, because environmental factors such as light, temperature and food availability often vary during the 24 h day, which can, in turn, influence an animal's decision to adjust their timing of activity (reviewed in [9–11]). Consequently, changes in abiotic conditions and in food availability may confound the effects of competition on timing of activities. The hypothesis that time-shifting reduces exploitation competition predicts that asynchronously active species pairs (e.g. diurnal versus nocturnal) should have a lower diet overlap than synchronously active species (i.e. a pair of diurnal or a pair of nocturnal species). However, studies on raptors [3] and lizards [12] failed to find support for this prediction. In non-endothermic species such as lizards, the authors later found that body size-related thermoregulatory constraints alone may explain the variation in activity time [13]. In the case of raptors, a subsequent paper suggested that interference competition may play a role in time partitioning [4].

An animal's ability to explore the opposite temporal niche compared to its typical active time may be evolutionarily constrained [9,14,15], because adaptation to a certain temporal niche (e.g. day or night) may require a set of morphological, physiological and behavioural changes (e.g. eye morphology, visual and other sensory systems and thermoregulatory ability; [6,14,16]). Nevertheless, groups of related species are ideal to test the importance of ecological drivers on variation in the timing of activity [17,18]. Various studies have attempted to explain why closely related species differ in the time when they are active, but few have focused on competition-related mechanisms (e.g. [15,19,20]).

The aim of this study was to investigate the relative importance of interference and exploitation competition as a selective force on time niche partitioning and specifically on the occurrence of time-shifting. To this end, we conducted a global scale comparative analysis on day- and night-active avian predators. Avian predators are suitable study objects in this context, because they (i) belong to three distinct phylogenetic orders (Accipitriformes, Falconiformes and Strigiformes), (ii) are functionally homogeneous (i.e. avian predators kill for food and smaller predator species are included as potential prey; [21,22]), (iii) are distributed over a wide range of biogeographical regions and (iv) vary in time when active (both between and within orders). Moreover, among raptors, interference competition is important: agonistic interactions occur frequently and can have stressful or even lethal consequences (e.g. harassment, loss of prey and predation; [4,21–23]), and hence strong negative fitness effects [24].

To study the evolution of time-shifting, we used the following general approach. (i) We collected information about activity times of avian predators and defined which species are time-shifters based on the ancestral state of the timing of activity for each order. (ii) We analysed the occurrence of time-shifting at a global level, using groups of avian predators that occupy a common geographical region (i.e. an assemblage) as the unit of analysis. This approach emphasizes the actual ecological background of a set of species. In each assemblage community, we considered the number of time-shifted species as an indicator of the (past) strength of selection on time partitioning. (iii) For each assemblage, we quantified the level of exploitation competition experienced by avian predators (currently or in the evolutionary past) as the total number of all sympatric avian predator species. This is reasonable because higher predator species richness implies fewer food resources per predator [25]. Similarly, we quantified the level of interference competition as the number of all sympatric avian predator species that are active during the period typical for the focal order. (iv) We then tested whether the level of exploitation and interference competition predicted between-assemblage variation in the number of time-shifted species.

Because the effect of competition can be mediated by the availability of the resource [2,26,27], we included the available time resource (i.e. day or night length) in the models. We also included body size in the model, because the outcome of direct interactions is typically size-dependent [28–30]. First, smaller species are more likely to lose competitive interactions and empirical studies on both prey and predator groups have shown that small species avoid interference competition by shifting their time of activity and avoiding encounters [10,31]. Thus, we predicted that time-shifters are typically smaller than other species from the same order in the assemblage. Second, dominant (i.e. larger) individuals benefit from foraging when others forage, because they can steal food from subdominant individuals [32]. Hence, we also expected to find fewer time-shifters among the large avian predators when (i) the total predator species richness in the assemblage is higher (because there are more species to steal food from or to kill and eat) and (ii) when the duration of the typical time resource (i.e. either day or night length) is longer (i.e. when there is more of the resource ‘time’ available).

## 2. Material and methods

### (a) Avian predator data

For all avian predator species of the world, except the vultures, because they typically feed on carcasses and rarely kill healthy animals, we compiled data on the period of activity and body size from the standard ornithological literature (see electronic supplementary material for details). To create maps and for assemblage-level analysis, we used the breeding range distribution of each species (i.e. the geographical extent of occurrence during the reproductive season), based on the study by Valcu *et al.* [33]. Because the breeding range distribution of avian predators is generally better described than the wintering grounds and because there are few strictly migratory species (48 out of 376; see electronic supplementary material, table S1), we did not include the wintering range in the analyses.

**Figure 1.**
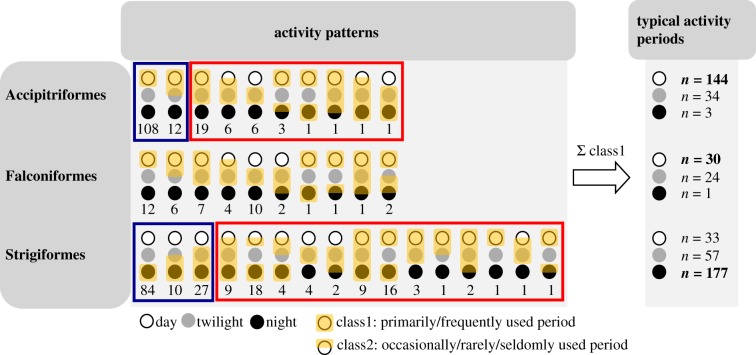
Number of species with different activity patterns, definitions of typical activity periods and typical/time-shifted species in the Accipitriformes, Falconiformes and Strigiformes. Day, twilight and night are depicted as open, grey and black circles, respectively. A circle overlaid by yellow shading indicates the primarily/frequently used period of activity (i.e. class 1); a half-circle overlaid by yellow shading indicates an occasionally/rarely/seldomly used period of activity (i.e. class 2). Numbers under each set of circles (different types of activity periods) indicate the number of species in each order that show the specific activity pattern. Note that all species that were scored as both diurnal and nocturnal (cathemeral) were most likely also active during twilight, but this might not have been specifically mentioned in the literature. The overall usage of activity periods for each order was calculated by counting the number of species that were primarily/frequently active (i.e. the number of fully overlaid circles by yellow shading) in each of the day, twilight and night periods. The typical activity period for each order is depicted in bold. Typical species (with respect to activity patterns) are framed in blue, while time-shifted species are framed in red. See Material and methods for further details.

Data on the period of activity were available for 396 species (192 Strigiformes, 158 Accipitriformes and 46 Falconiformes). First, we assigned each species to one or more of three categories: day-active (described as active during the daytime), night-active (described as active during the night) and twilight-active (crepuscular, active during twilight until dawn or dusk). Second, for each of these three active period categories, we scored for each species whether it is a primarily/frequently used period (class 1) or an occasionally/rarely/seldomly used period (class 2) (figure 1). When a direct description of the active period was unavailable (*n* = 156 species), we assigned the active period based on descriptions of related behaviours such as roosting (i.e. resting and sleeping behaviour) and foraging, whereby we assumed that if the species showed a similar foraging tactic to other day- or night-active species, it had a similar active time (i.e. for Accipitriformes and Falconiformes, foraging by soaring and hovering was categorized as day-active; for Strigiformes, foraging by perching was categorized as night-active). When this information was also missing (*n* = 17 species), we assigned the active period based on eye morphology [18].

Body size was defined as total body length, measured from the tip of the bill, or the edge of the head in the case of Strigiformes, to the end of the tail of a flattened specimen. Data were available for all 498 species (228 Accipitriformes, 206 Strigiformes and 64 Falconiformes). If instead of the typical body size value, an interval (minimum and maximum) was given for a species, we calculated the average of the minimum and maximum values. If the minimum and maximum values of a species were not indicated, we calculated the average of all entries available for that species (e.g. average, minimum or maximum values for either males or females).

### (b) Ancestral state reconstruction of the typical activity period

We first defined the typical period of activity for all species belonging to one of three independent, monophyletic orders: Accipitriformes, Strigiformes and Falconiformes [34,35]. For each order, we determined the ancestral state of the activity pattern using the R package ‘ape’ v. 4.1 [36], run in R v. 3.4.3. (R Development Core team). Based on the earlier assignment into the three activity categories, we further classified all Accipitriformes and Falconiformes species as either strictly diurnal, i.e. exclusively day-active (*n* = 120 species), or not (*n* = 38 species). Species belonging to the Strigiformes were classified as either strictly nocturnal, defined as exclusively night-active (*n* = 84 species) or active during the night and during twilight (*n* = 37 species), or not (*n* = 71 species) (figure 1). We included Strigiformes species that are active during twilight into the strictly nocturnal group, while we did not include twilight-active Falconiformes and Accipitriformes in the strictly diurnal group, for three reasons. Firstly, birds evolved from diurnal dinosaurs [18,37]; secondly, twilight periods are short and characterized by lower light levels, such that nocturnal activity is often described with dawn and dusk included (e.g. in small mammals; [38]); thirdly, for birds, shifting from night-active to day-active is presumably easier than the other way around. Thus, we considered activity type as a discrete trait with two states: strict type and non-strict type. We then ran ancestral state estimations for each order, based on 9999 trees from the published bird tree database [34] and applying a model with maximum-likelihood estimation and an equal evolution rate. The ancestral state (strict or not) was determined as the type with the highest posterior probability at the basal position.

Among the Accipitriformes and the Falconiformes, 144 out of 158 species (91%), respectively, 30 out of 46 (65%) were considered day-active (including both strictly and not strictly day-active species), whereas among the Strigiformes, 177 out of 192 species (92%) were considered night-active (including both strictly and not strictly night-active species, see electronic supplementary material). The ancestral state reconstruction shows that at the basal position, Accipitriformes are strictly diurnal, while Strigiformes are strictly nocturnal (electronic supplementary material, figure S1). However, the analysis showed that Falconiformes have a mixed ancestry of both strictly diurnal and non-strictly diurnal (i.e. crepuscular) species (electronic supplementary material, figure S1).

### (c) Identification of time-shifters

We defined time-shifted species as those that are active outside of the period typical for species belonging to the same order. We decided *a priori* to use only orders for which the ancestral state was either strictly diurnal or strictly nocturnal, because this allowed unequivocal identification of the direction of the shift in the timing of activity, and hence a clear-cut definition of time-shifting species. Therefore, we excluded the Falconiformes from the analyses of time-shifters.

Species belonging to the Accipitriformes were rarely truly nocturnal, but 34 species have been reported as active during twilight and four as occasionally active during the night (see electronic supplementary material). We considered all these species (*n* = 38, 24% of all Accipitriformes) as time-shifters, even though they were also active during the day.

For the Strigiformes, species have been reported as day-active (*n* = 33, among which most are also active during the night, i.e. cathemeral), occasionally active during the day (*n* = 32) or exclusively crepuscular (*n* = 6; see electronic supplementary material). So, for this order, we defined time-shifters as those species that were not strictly night-active (71 species, 37% of all Strigiformes).

### (d) Measures of exploitation and interference competition pressure

To estimate the intensity of competition, we considered all 498 avian predators of the three orders. First, we overlaid the earth's surface with a grid with cell size 112.5 × 112.5 km. Each cell is thus a well-defined geographical area, which we refer to as an assemblage. We then determined for each cell of the grid all the species whose breeding range overlapped that cell.

For each assemblage, we quantified the exploitation competition pressure on a species as the total predator species richness in that assemblage, i.e. all sympatric avian predators. We quantified the interference competition pressure on a focal species as the number of all day-active predator species (including day-active Strigiformes and day-active Falconiformes) if the focal species belonged to the Accipitriformes and the number of all night-active species (including night-active Accipitriformes and night-active Falconiformes) if it belonged to the Strigiformes. A predator species was included, regardless of the order it belonged to, if it had been categorized as frequently active during the period typical for the focal order (class 1, see above). We also quantified the interference competition pressure for a subset of birds, namely the large Strigiformes (body size > 30 cm, see Results). For this subgroup, we included only avian predator species larger than 30 cm, assuming that smaller species would not compete with larger ones through direct interference [39].

### (e) Measures of the typical time resource

We determined the duration of the typical time period available to each species (thus, for time-shifters, the available time had they not shifted) based on latitudinal variation in day/night length. Thus, for Accipitriformes, we used day length (i.e. the period from sunrise to sunset, when the centre of the solar disc is above the horizon), while for species belonging to the Strigiformes, we included the night length (i.e. the period from dusk to dawn, when the centre of the solar disc is less than 6° below the horizon). For each assemblage, day and night length at summer solstice were calculated using ‘maptools’ v. 0.9-2 [40] as a proxy for the available typical time resource during the breeding season.

### (f) Mapping and statistical analyses

Mapping and all statistical analyses were carried out in R v. 3.4.3. Body size was log_10_ transformed to correct for skewness. Visual inspection of the distribution of body size in the Strigiformes suggested bimodality. We formally tested for bimodality in body size using the R package ‘diptest’ v. 0.75-7 [41].

Body size comparisons between time-shifted species and the typical species in the same order, with and without control for phylogeny, were carried out using generalized least-squares (gls) models with the R package ‘nlme’ [42]. For phylogenetic informative models, we used Pagel's λ [43] as the measurement of the strength of the phylogenetic signal. We firstly estimated λ with regard to body size by fitting log_10_ body size for all Accipitriformes and Strigiformes species using 9999 trees with the function ‘phylosig’ in the R package ‘phytools’ (v. 0.6-44) [44]. We then used the tree with the median λ value as the average phylogeny for body size analysis at both species and assemblage levels. We calculated means and 95 percentiles of the fixed effects and of Pagel's λ [43], based on 9999 trees from the published bird tree database [34] to account for uncertainty.

Mapping and assemblage-level analyses were performed using ‘rangeMapper’ 0.3-1 [33]. We created a rangeMapper project using a grid with cell size of 112.5 × 112.5 km on a Mollweide projection. In total, we thus obtained 10 538 grid cells that were further classified into 11 zoogeographical realms [45]. The rangeMapper project included the species' breeding range distributions, the zoographical realm ranges and life-history data. Maps of species richness (i.e. the number of time-shifters and the two competition pressure indices), the duration of the typical time niche available to time-shifters (i.e. day or night length) and the difference in body size between time-shifters and typical species competitors (absolute body size differences and body size differences after controlling for phylogeny) were generated within the rangeMapper project. For phylogenetic informative models of body size comparisons, we used gls models from the R package ‘nlme’ [42], by including the average phylogeny of the species set in the assemblage into the model.

We calculated the time-shifter species richness simply by counting the number of time-shifted species in each assemblage. For a particular assemblage, time-shifter species richness was only counted as zero when the total species richness of either Accipitriformes or Strigiformes was larger than zero, but no time-shifted species of that respective order occurred. Similarly, for large and small Strigiformes (body size larger or smaller than 30 cm, see Results), we only assigned time-shifter species richness as zero for assemblages where large, respectively, small Strigiformes occurred.

We analysed the time-shifted species richness for separate orders or subgroups using ‘lme’ models in the ‘nlme’ package. For each model, we included the indices for the level of interference and exploitation competition, as well as the duration of the typical time period as fixed effects. We also fitted the zoogeographical realm [45] of the assemblage as a random intercept. This was done because for terrestrial vertebrates each zoogeographical realm can be considered as an independent unit of speciation [46]. To avoid strong colinearity between the predictors, predator species richness was included as a random slope within each realm. To model spatial autocorrelation, and hence to account for ‘pseudo-replication’, we used a spatial correlation structure within each zoogeographical realm. Initially, we tested different spatial autocorrelation structures and selected the one with the lowest model AIC (Akaike information criterion) value (electronic supplementary material, table S2). This was an exponential correlation structure ‘corExp’ that included a nugget effect, which accounts for larger than expected variance of closely located assemblages. To keep computing time within reasonable limits, we ran the above models after randomly sampling—within each zoogeographical realm—40% of all assemblages where avian predators occur. We thus obtained *n* = 4036 assemblages for models of Accipitriformes and Strigiformes, *n* = 3447 for models of small Strigiformes, and *n* = 4020 assemblages for models of large Strigiformes. All response variables and fixed effects were scaled (*z*-scored) to allow comparison of estimated effect sizes. Confidence intervals (95%) of fixed effects were calculated using the ‘glht’ function from the R package ‘multcomp’ v.1.4-8 while controlling for multiple testing [47].

## 3. Results

### (a) Body size distribution of time-shifted species

**Figure 2.**
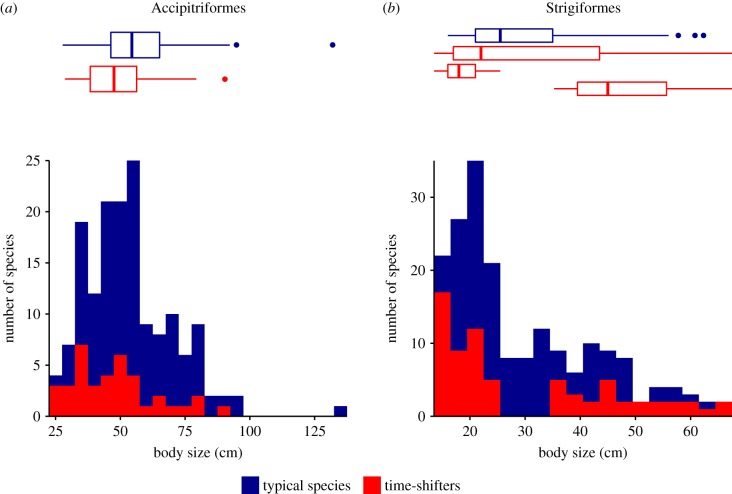
Body size distributions of time-shifters and typical species in (*a*) Accipitriformes (typical species: *n* = 120; time-shifter species: *n* = 38) and (*b*) Strigiformes (typical species: *n* = 121, all time-shifter species: *n* = 71, large time-shifter species: *n* = 28, small time-shifter species: *n* = 43). Shown are box plots and histograms for typical species (blue).

The body size distribution of Accipitriformes time-shifter species is unimodal (bimodaility test: *D* = 0.052, *p* = 0.61, *n* = 38; figure 2*a*). Time-shifters are significantly smaller than the non-time-shifted species of the same order (*p* = 0.02; figure 2*a*; electronic supplementary material, table S3). However, this size difference disappears after accounting for phylogeny (*p* = 0.87; electronic supplementary material, table S4).

The body size distribution of Strigiformes time-shifted species is clearly bimodal (*D* = 0.067, *p* = 0.014, *n* = 71), with the lowest frequency at a body size of 30 cm (figure 2*b*). Therefore, we classified Strigiformes time-shifters into two groups: large species (greater than 30 cm) and small species (less than 30 cm). The small time-shifters are indeed smaller than the sympatric typical (night-active) Strigiformes (*p* < 0.0001; figure 2*b*; electronic supplementary material, table S3), even when accounting for phylogeny (*p* = 0.03; electronic supplementary material, tables S4). The large time-shifters are indeed significantly larger than the sympatric typical species, even when taking phylogeny into account (*p* < 0.0001; figure 2*b*; electronic supplementary material, tables S3 and S4).

After accounting for phylogeny, Accipitriformes time-shifters are smaller than the sympatric non-time-shifted species in 68% of assemblages (4341 out of 6379; electronic supplementary material, figure S2*a*). When considering all Strigiformes, time-shifters are smaller than the sympatric, non-time-shifted species in 63% of assemblages (4472 out of 7051; electronic supplementary material, figure S2*b*). However, we also partitioned Strigiformes time-shifters into a small and a large subgroup. Small Strigiformes time-shifters are indeed smaller than their sympatric, non-time-shifted relatives in 99% of assemblages (5020 out of 5084; electronic supplementary material, figure S2*c*), while large time-shifters are indeed larger than the sympatric non-time-shifters in 81% of assemblages (5576 out of 6848; electronic supplementary material, figure S2*d*).

### (b) Global distribution of time-shifted species

**Figure 3.**
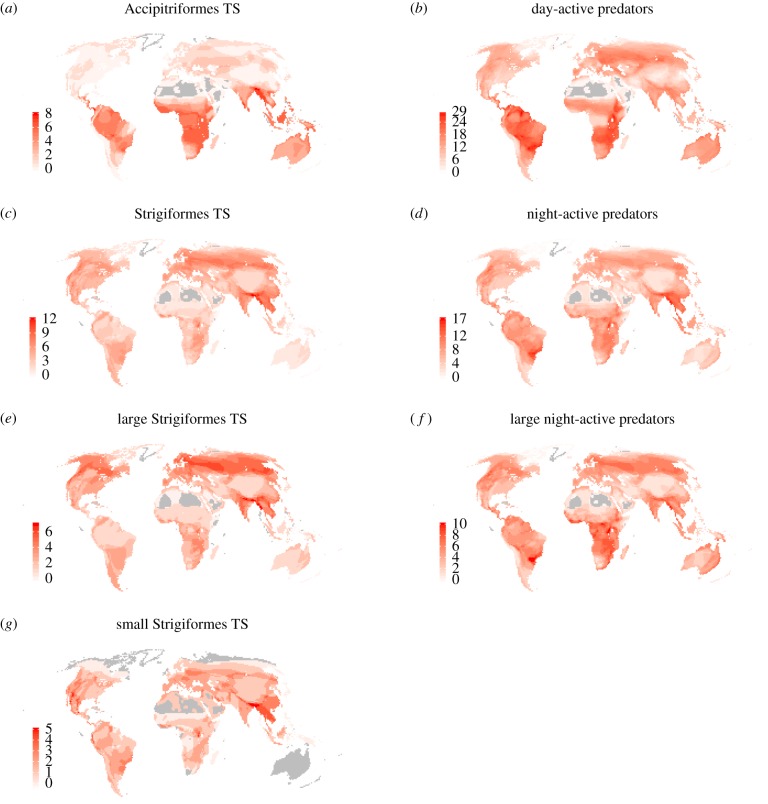
Global distribution of the number of species (i.e. species richness) for (*a*) Accipitriformes time-shifters (TS), (*b*) day-active (avian) predator species, i.e. an index for interference competition pressure for Accipitriformes time-shifters, (*c*) Strigiformes time-shifters, (*d*) night-active predator species, i.e. an index for interference competition pressure for all Strigiformes and for small Strigiformes time-shifters, (*e*) large Strigiformes time-shifters (body size > 30 cm), (*f*) large night-active predator species (body size > 30 cm), i.e. an index for interference competition pressure for large Strigiformes time-shifters, and (*g*) small Strigiformes time-shifters (body size < 30 cm). Increasing intensity of red reflects higher species richness. Grey indicates areas that lack the relevant avian predator species.

The number of time-shifted species varies geographically (figure 3). In the Accipitriformes, more time-shifted species are found near the equator and in the Southern Hemisphere (figure 3*a*), also compared with the total number of day-active predators (figure 3*b*).

Overall, the number of time-shifted Strigiformes was higher in northern latitudes (figure 3*c*), also compared with the total number of night-active predators (figure 3*d*). This effect was mainly seen in the large Strigiformes (figure 3*e*,*f*). Small time-shifter Strigiformes were absent in Oceania and in arctic and subarctic regions (figure 3*g*).

Overall, time-shifted species from both orders appear to be more frequent in the Oriental zoogeographical realm (figure 3*a*,*c*,*e*,*g*).

### (c) Explaining variation in the number of time-shifters

**Figure 4.**
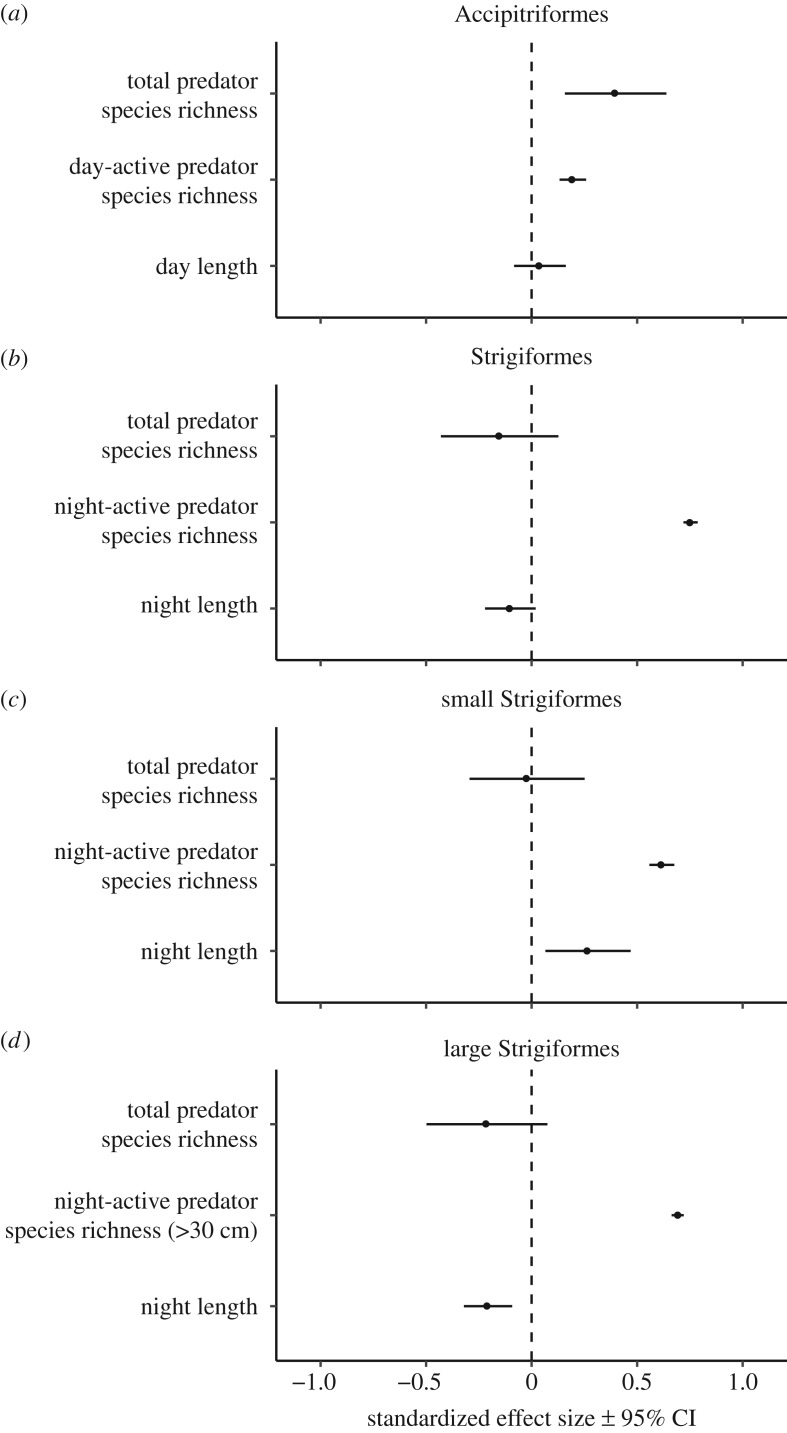
Predictors of time-shifter species richness in each assemblage for (*a*) Accipitriformes (model based on 4036 assemblages), (*b*) all Strigiformes (model based on 4036 assemblages), (*c*) small Strigiformes (model based on 3447 assemblages), and (*d*) large Strigiformes (model based on 4020 assemblages). Shown are estimated effect sizes for each predictor (scaled) with their 95% confidence intervals (see Material and methods and electronic supplementary material, table S5 for model details). Total predator species richness, i.e. an index for exploitation competition pressure, was estimated as the total number of sympatric avian predator species in the assemblage. Day-/night-active predator species richness, i.e. an index of interference competition pressure, was estimated for Accipitriformes, Strigiformes and small Strigiformes as the total number of avian predator species in the assemblage that are active during the period typical for the order to which the focal species belongs; for large Strigiformes, it was estimated as the number of large night-active species (body size > 30 cm). Local day or night length (at summer solstice) reflects the available time resource in the absence of time-shifting in each assemblage.

For the Accipitriformes, the number of time-shifted species in an assemblage is explained by the total number of predator species (i.e. by exploitation competition pressure) and by the number of day-active predator species (i.e. by interference competition pressure; figure 4*a*; electronic supplementary material, table S5). Local day length (i.e. the available typical time resource) has no effect on the number of time-shifters (figure 4*a*; electronic supplementary material, table S5).

The total number of time-shifted Strigiformes species increases with increasing numbers of night-active predator species (i.e. with increasing interference competition pressure), but is independent of the total number of predator species in the assemblage (i.e. independent of exploitation competition pressure; figure 4*b*; electronic supplementary material, table S5). The overall species richness of time-shifted Strigiformes was also independent of the availability of the typical time resource (i.e. no effect of night length, figure 4*b*; electronic supplementary material, table S5).

When modelling the large and small Strigiformes species separately, a somewhat different picture emerges. For the small Strigiformes, the number of time-shifted species is only explained by the number of night-active predator species in the assemblage (i.e. by interference competition pressure; figure 4*c*; electronic supplementary material, table S5). However, contrary to the expectation, there are also more time-shifted species when more of the typical time resource is available (i.e. a positive effect of night length, figure 4*c*; electronic supplementary material, table S5). For the large Strigiformes, assemblages have more time-shifted species with an increasing number of night-active predator species and with decreasing availability of the typical time resource (i.e. negative relationship with night length, figure 4*d*; electronic supplementary material, table S5).

## 4. Discussion

Shifting the time of activity is found more frequently among the Strigiformes (37% of species) than among the Accipitriformes (24% of species). In the Strigiformes, the shift in the timing of activity was also stronger: although some Strigiformes time-shifters were only crepuscular, there are many day-active time-shifter species in this group, whereas for the Accipitriformes time-shifters were seldomly active at night. Among the time-shifted Accipitriformes, only the letter-winged kite, *Elanus scriptus*, is considered a nocturnal species [48], while all others are species that are most active during the twilight period. These observations imply that the daily timing of activity is a plastic trait, at least in avian predators, and suggest that it is easier for typically night-active species to shift their activity into the day than for day-active species to become active at night.

Overall, our results provide evidence for a role of competition in explaining the evolution of time-shifting. We considered effects of (i) body size (as a proxy for competitiveness), (ii) day or night length (as a proxy for the available time resource), (iii) the total number of avian predators in the assemblage (as a proxy for exploitation competition pressure), and (iv) the total number of avian predators in the assemblage that are active during the period typical for a given order (as a proxy for interference competition pressure). Below, we discuss the importance and the direction of these effects for each order.

Our results suggest that interference competition has the most consistent effect on the number of time-shifter species in an assemblage: all models show that assemblages with a higher number of direct competitors (i.e. more species that are active during the typical period for that order) contain more time-shifters. The direction of this effect is the same for the two orders and for large and small time-shifted Strigiformes (figure 4; electronic supplementary material, table S5). When the migratory species are excluded from the analysis or when using the same definition for typical Strigiformes species (i.e. including only explicitly nocturnal species as typical species), the effect stays the same (electronic supplementary material, figures S3, S4 and tables S6, S7).

In animals, body size is one of the key indicators predicting the outcome of contests [28–30,49,50]. Among avian predators, individuals of smaller species are not only less likely to win interactions over food, but they are also more likely to become prey items themselves [24,28]. Given that interference competition can have such strong negative fitness effects, smaller species may benefit more from shifting their active period to avoid such interactions. Our results indeed support—to some extent—that smaller species are more likely to become time-shifters. In the Accipitriformes, time-shifters were smaller than typical species in the majority of assemblages and overall this effect was significant (figure 2*a*; electronic supplementary material, table S3). For the Strigiformes, the results show a more complex pattern: the majority of time-shifters (61%) were relatively small compared with the typical species, but there is also a group of relatively large time-shifters (bimodal distribution, figure 2*b*).

Our results suggest that when species face a high probability of interaction (competition) with other avian predators during the typical active period, more species, and especially the small ones, shifted their active time away from the typical time period. In classical textbooks, mammals are described as small nocturnal insectivores in their early evolutionary history [6,8], while birds are described as small diurnal carnivorous dinosaurs [18,37]. The evolution of nocturnality in mammals has been discussed in the light of avoiding direct competition with and predation by the dominant (large) diurnal dinosaurs in the Jurassic [8,51,52]. Our findings support the suggestion derived from the nocturnal bottleneck hypothesis that (interference) competition serves as a strong selection pressure driving the evolution of nocturnality in typically day-active predators.

Another factor that can influence the strength or importance of interference competition is the amount of the resource ‘time’ that is available. If the typical period of activity, i.e. day or night length, is longer, individuals operating within this period will have more opportunity (time) to avoid direct interference. Hence, we expect fewer time-shifters when days, respectively nights, are longer. Our results show mixed evidence for this hypothesis. In the Accipitriformes, day length did not affect the number of time-shifted species, perhaps because in many of these species foraging is limited to or most efficient at certain periods of the day (e.g. soaring flight takes advantage of warm, rising air masses heated by the sun [53] and hunting by hovering occurs more often during periods of moderate wind) [54,55]. For the small Strigiformes, where we expect interference competition pressure to be strongest, the duration of the night had a counterintuitive effect, i.e. we found more time-shifted species when nights were longer (figure 4*c*; electronic supplementary material, table S5). However, this effect was not particularly robust: it was absent when migratory species were excluded (see electronic supplementary material, figure S3 and table S6). This suggests that the positive effect of the duration of the night might be due to the inclusion of a few strictly migratory small Strigiformes, i.e. small, time-shifted Strigiformes migrate to regions where the night is longer for breeding. For the large Strigiformes, the effect was in the expected direction: the longer the night, the fewer time-shifted species in an assemblage (figure 4*d*; electronic supplementary material, table S5). A theoretical study showed that dominant (larger) animals can benefit from foraging together with other species [32]. Thus, it might be more beneficial for the large Strigiformes to become active outside of the typical activity period when this resource is limited, i.e. when nights are short. However, the effect also disappeared when migratory species were excluded (electronic supplementary material, figure S3 and table S6).

We also found some support for an effect of exploitation competition pressure on the number of time-shifted species, but this effect was less consistent between orders. A higher total avian predator species richness (all species included, independent of when they were active) was associated with a larger number of time-shifted species in the Accipitriformes (figure 4*a*; electronic supplementary material, figure S3*a* and tables S5, S6). But, exploitation competition pressure did not significantly predict the number of small or large Strigiformes time-shifted species (figure 4*b–d*; electronic supplementary material, figures S3*b*–*d*, S4*b*–*d* and tables S5–S7).

In this study, we considered the typical activity time for an order to be day or night in a broad sense. Of course, species could also partition the time when they forage or engage in other activities within the day or night. Therefore, considering the total number of avian predator species that are active during the day or night might overestimate the actual effect of interference competition for certain communities. On the other hand, we used two orders as independent replicates and sampled 40% of all assemblages worldwide. The resulting models show an overall strong positive effect of interference competition pressure on how many species shifted their activity time. Because of the nature of comparative analyses, our results do not ‘prove’ that exploitation or interference competition pressure is causally related to the number of time-shifted species. Indeed, we cannot exclude a role of speciation: once a single ancestral species evolved the ability to fully explore a different time, further partitioning of food and habitat resources may have led to a radiation into different species. For example, out of the 43 small time-shifted Strigiformes, 27 belong to the genus *Glaucidium*; similarly, 21 out of the 28 large time-shifters are closely related species.

In summary, our results simply demonstrate an association between time-shifted species richness and its potential evolutionary drivers, thus shedding light on possible mechanisms behind the evolution of time-shifting. Our study cannot exclude other possible drivers of time-shifting, such as temperature, habitat characteristics or other interspecific interactions, which might be important for understanding local community structure. As noted earlier, in different communities, animals may partition resources in different ways [2]. Future studies could include additional indicators of the probability of interference and exploitation competition in particular communities, for example by considering the actual diet of the different predator species as well as information about population densities.

## 5. Conclusion

Taken together, our study clearly supports the interference competition hypothesis, suggesting that animals partition their timing of activity to reduce the costs associated with direct interference competition. Although phylogeny constrains animals' abilities to shift activity time, our results highlight the importance of interference competition as an evolutionary driver of time-shifting and suggest a role of body size in mediating the mechanism of time partitioning.

## Supplementary Material

ESM

## Data accessibility

Electronic supplementary material and R scripts can be found in the Open Science Framework at https://osf.io/2rfm7/.
