# Supplementary material for: Interference competition pressure predicts the number of avian predators that shifted their timing of activity
Source: Proc Biol Sci. 2018 Jun 6;285(1880):20180744. doi: 10.1098/rspb.2018.0744 (PMC6015849; doi:10.1098/rspb.2018.0744)
Supplement: ESM [file rspb20180744supp1.docx]

**Electronic Supplementary Materials**

**Interference competition pressure predicts the number of avian predators that shifted their timing of activity**

**Yifan Pei**^1^**, Mihai Valcu**^1^**, Bart Kempenaers**^1*^

^1^ Department of Behavioural Ecology and Evolutionary Genetics, Max Planck Institute for Ornithology, 82319 Seewiesen, Germany

Keywords: time partitioning, interference competition, exploitation competition, species richness, Accipitriformes, Strigiformes, predators, body size

Figures & Tables: 4 figures

Supplementary materials: 4 figures, 8 tables

Address for correspondence:

**Bart Kempenaers**^*^, Department of Behavioural Ecology and Evolutionary Genetics, Max Planck Institute for Ornithology, Eberhard-Gwinner-Str. 7, 82319 Seewiesen, Germany, Phone: 0049-8157-932334

Email: [b.kempenaers@orn.mpg.de](mailto:ypei@orn.mpg.de)

Supplementary materials**:** Figures S1-S3, Tables S1-S7.

**
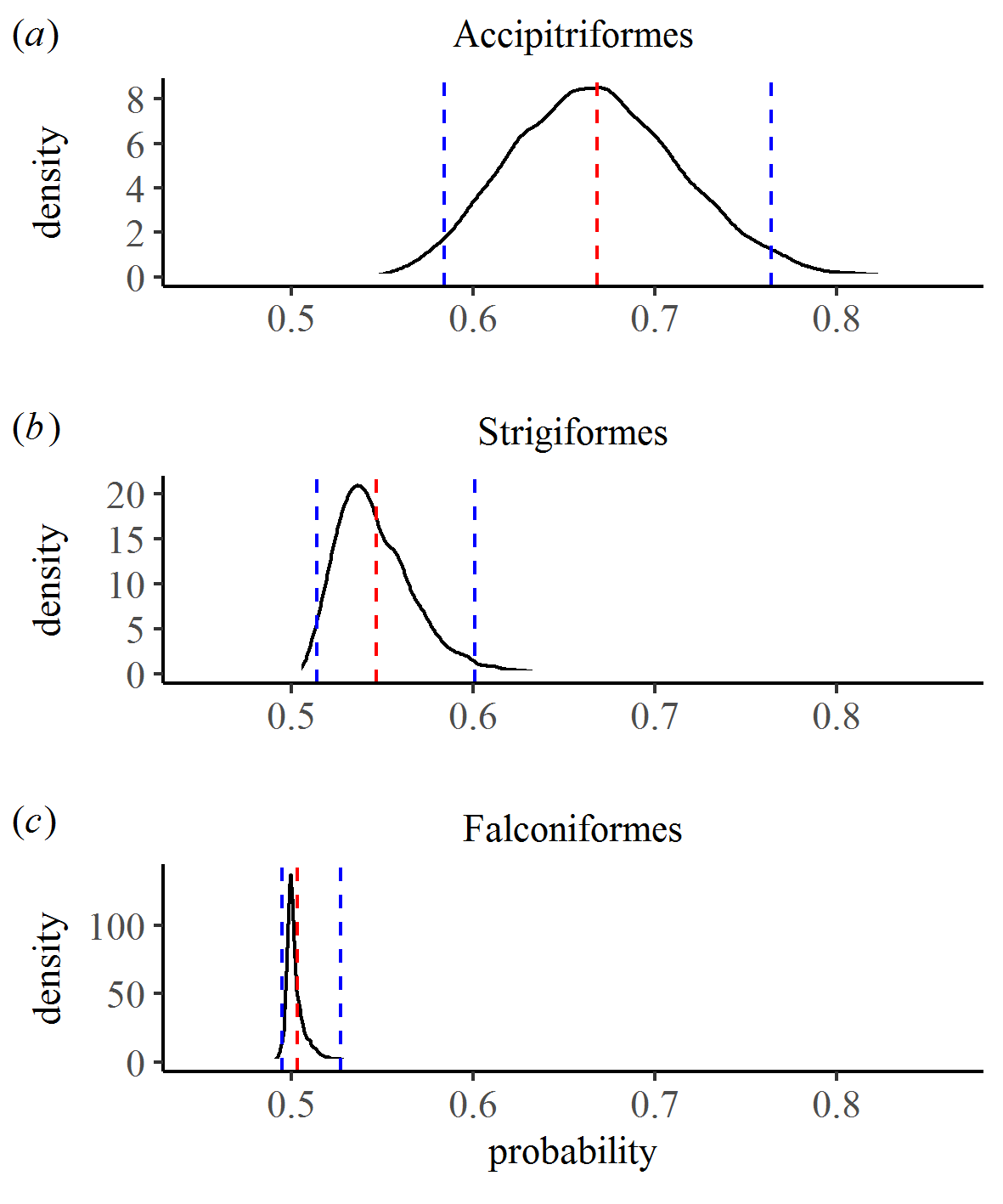
**

**Figure S1.** Ancestral character state estimates of strictness of activity pattern for the typical active time for (*a*) Accipitriformes, (*b*) Strigiformes and (*c*) Falconiformes. For Accipitriformes and Falconiformes, the estimated distribution indicates the probability of the ancestral state to be strictly diurnal; for Strigiformes, the estimated distribution indicates the probability of the ancestral state to be strictly nocturnal. The dashed red lines indicate the estimated mean probability, and the dashed blue lines indicate the 2.5% and 97.5% percentiles, respectively. The probabilities were estimated based on 9999 trees for each order. The mean probability distribution of Accipitriformes is 0.668 (2.5% percentile = 0.584 and 97.5% percentile = 0.764) to be strictly diurnal, the mean probability distribution of Strigiformes is 0.547 (2.5% percentile = 0.514 and 97.5% percentile = 0.601) to be strictly nocturnal and the mean probability distribution of Falconiformes is 0.503 (2.5% percentile = 0.495 and 97.5% percentile = 0.527) to be strictly diurnal.


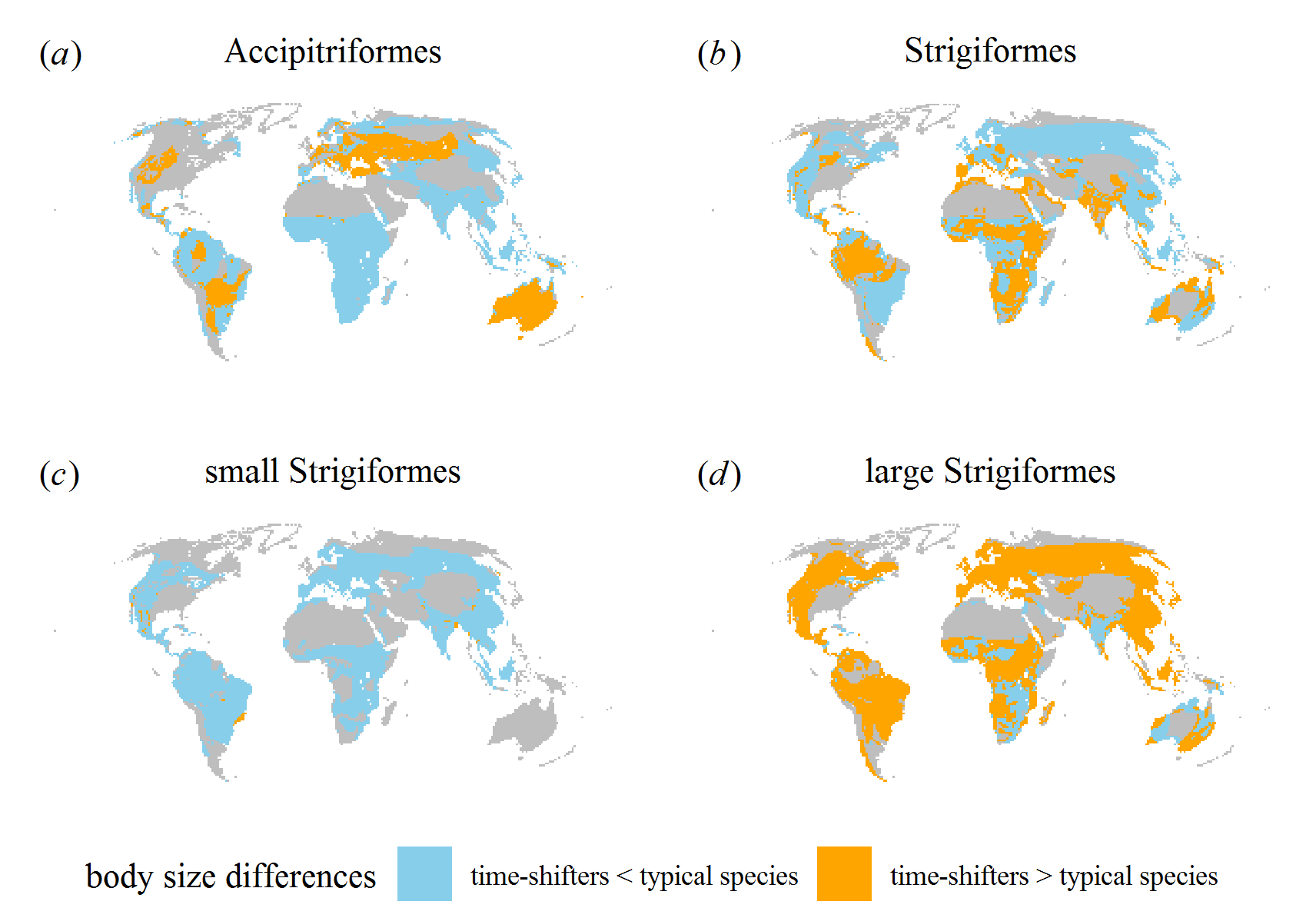


**Figure S2**. Global maps of body size differences between time-shifters and the typical (non-time shifted) species for (*a*) Accipitriformes, (*b*) all Strigiformes, (*c*) small Strigiformes and (*d*) large Strigiformes. Body size differences were estimated while controlling for phylogeny running a species-level model within each assemblage using the average phylogeny. Assemblages (grid cells, see Methods) where time-shifters are smaller than the typical species are depicted in blue, assemblages where time-shifters are larger are depicted in orange. Areas where time-shifter species or avian predator species are absent are depicted in grey.


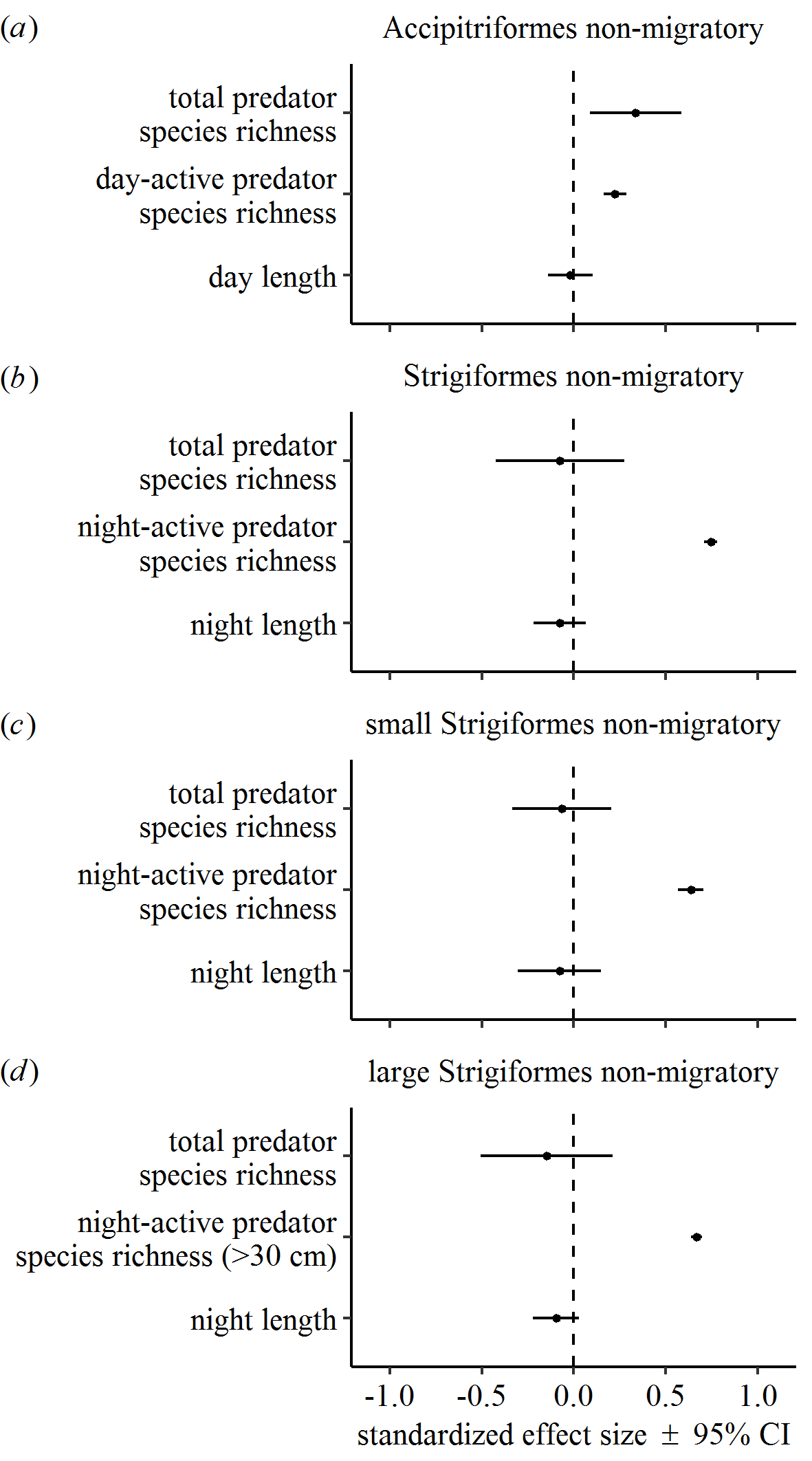


**Figure S3.** Predictors of time-shifter species richness in each assemblage from models excluding strictly migratory species for (*a*) Accipitriformes (model based on 3949 assemblages), (*b*) all Strigiformes (model based on 3849 assemblages), (*c*) small Strigiformes (model based on 3054 assemblages) and (*d*) large Strigiformes (model based on 3810 assemblages). Shown are estimated effect sizes for each predictor (scaled) with their 95% confidence intervals (see Methods and table S6 for model details). Total predator species richness, i.e. an index for exploitation competition pressure, was estimated as the total number of sympatric avian predator species in the assemblage. Day-/night-active predator species richness, i.e. an index of interference competition pressure, was estimated for Accipitriformes, Strigiformes and small Strigiformes as the total number of avian predator species in the assemblage that are active during the period typical for the order to which the focal species belongs; for large Strigiformes it was estimated as the number of large night-active species (body size >30 cm). Local day or night length (at summer solstice) reflects the available time resource in the absence of time shifting in each assemblage.


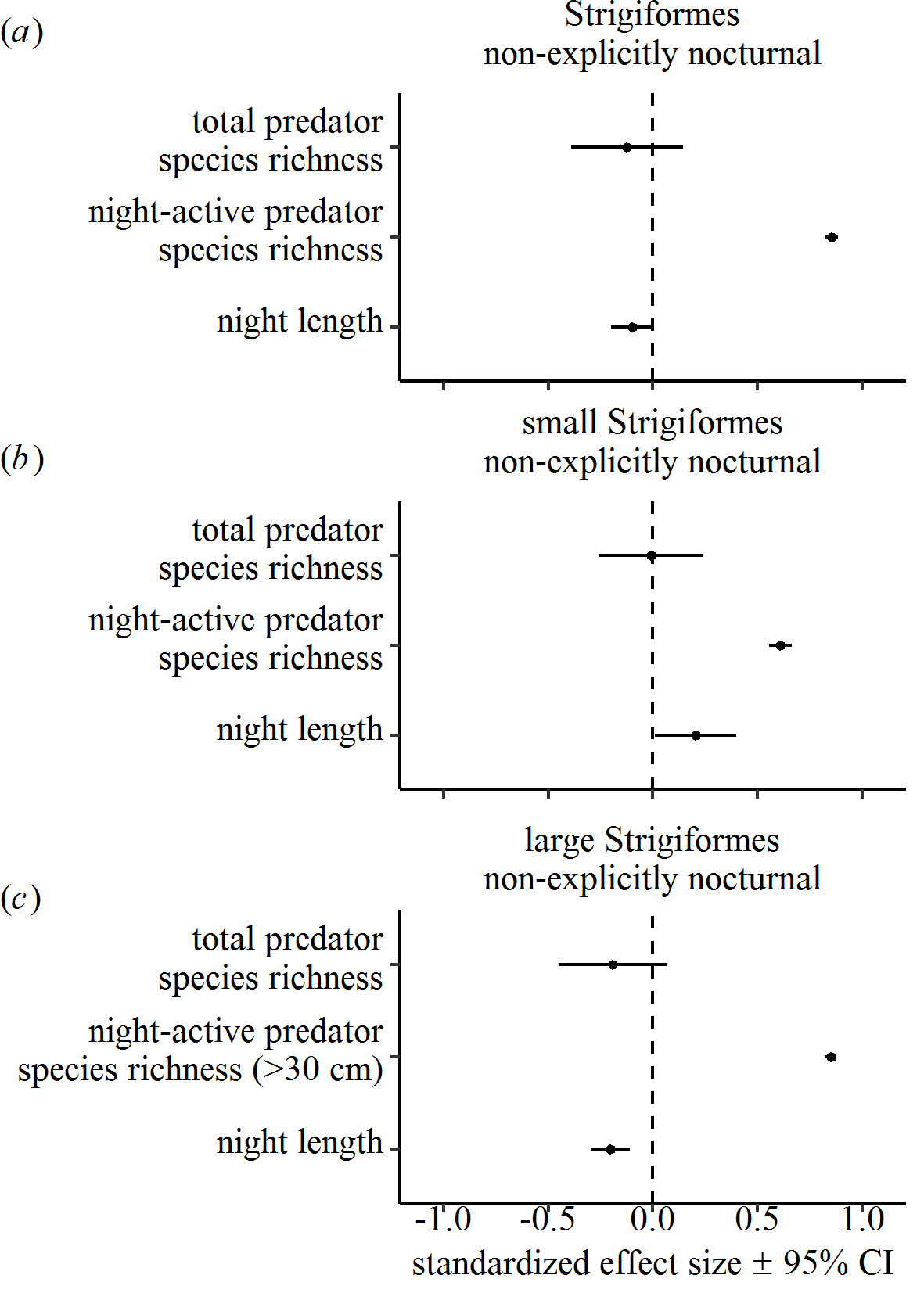


**Figure S4.** Predictors of time-shifter species richness in each assemblage from Strigiformes models using the same criteria for typical species as Accipitriformes (i.e. including only explicitly nocturnal species as typical species) for (*a*) all Strigiformes (model based on 4036 assemblages), (*b*) small Strigiformes (model based on 3447 assemblages) and (*c*) large Strigiformes (model based on 4020 assemblages). Shown are estimated effect sizes for each predictor (scaled) with their 95% confidence intervals (see Methods and table S7 for model details). Total predator species richness, i.e. an index for exploitation competition pressure, was estimated as the total number of sympatric avian predator species in the assemblage. Day-/night-active predator species richness, i.e. an index of interference competition pressure, was estimated for Accipitriformes, Strigiformes and small Strigiformes as the total number of avian predator species in the assemblage that are active during the period typical for the order to which the focal species belongs; for large Strigiformes it was estimated as the number of large night-active species (body size >30 cm). Local day or night length (at summer solstice) reflects the available time resource in the absence of time shifting in each assemblage.

**Table S1.** Summay of the number of strictly migratory species and the number of resident, nomadic or partially migratory species among the Accipitriformes, Falconiformes and Strigiformes.

| **order** | **type** | **number of strictly migratory species** | **number of resident, nomadic and partially migratory species** | |
| --- | --- | --- | --- | --- |
| **Accipitriformes** | typical species* | 22 | | 62 |
|  | time-shifted species | 6 | | 23 |
|  | others^†^ | 1 | | 14 |
| **Falconiformes** | typical species* | 0 | | 14 |
|  | time-shifted species | 7 | | 15 |
|  | others^†^ | 0 | | 9 |
| **Strigiformes** | typical species^‡^ | 5 | | 114 |
|  | large-bodied time-shifted species^¶^ | 3 | | 25 |
|  | small-bodied time-shifted species^¶^ | 4 | | 39 |
|  | others^†^ | 0 | | 13 |

* Day-active.

^†^ Refers to avian predator species where data on the timing of activity are missing (not included in the analyses).

^‡^ Night-active.

^¶^ For definition, see Methods and figure 1, 2 in the main text.

**Table S2.** Model selection for correlation structure (see Methods for details). Models were based on 5% of the assemblages (N = 694 for Accipitriformes and Strigiformes, N = 690 for Large Strigiformes and N = 597 for Small Strigiformes). N_p_ is the number of parameters, AICc is the corrected Akaike’s Information Criterion [48], ΔAICc is the change in AICc. Models were selected based on the lowest AICc value; the selected model described in the main text, i.e. the exponential correlation structure with nugget effect, is indicated in bold.

| **Models** | **correlation structure** | **nugget effect included** | **N_p_** | **AICc** | **ΔAICc** |
| --- | --- | --- | --- | --- | --- |
| **Accipitriformes** | **exponential** | **yes** | **10** | **496.420** | **0.000** |
|  | spherical | yes | 10 | 508.580 | 12.160 |
|  | exponential | no | 9 | 530.481 | 34.060 |
|  | Gaussian | yes | 10 | 552.565 | 56.144 |
| **Strigiformes** | **exponential** | **yes** | **10** | **325.218** | **0.000** |
|  | spherical | yes | 10 | 337.694 | 12.475 |
|  | exponential | no | 9 | 365.610 | 40.392 |
|  | Gaussian | yes | 10 | 383.766 | 58.548 |
| **Small Strigiformes** | **exponential** | **yes** | **10** | **370.667** | **0.000** |
|  | exponential | no | 9 | 380.340 | 9.674 |
|  | spherical | yes | 10 | 382.515 | 11.848 |
|  | Gaussian | yes | 10 | 416.550 | 45.884 |
| **Large Strigiformes** | **exponential** | **yes** | **10** | **893.428** | **0.000** |
|  | spherical | yes | 10 | 900.272 | 6.844 |
|  | Gaussian | yes | 10 | 909.969 | 16.541 |
|  | exponential | no | 9 | 938.184 | 44.757 |

**Table S3.** Models of body size differences between time shifters and the typical, non-time shifted species from the orders Accipitriformes and Strigiformes. Shown are estimates with their 95% confidence intervals (CI).

| **model** | **predictor** | **estimate** | | **SE** | **lower**  **CI** | **upperCI** | **z** | **P** | **predictor correlations^*^** | | | |
| --- | --- | --- | --- | --- | --- | --- | --- | --- | --- | --- | --- | --- |
|  |  |  |  |  |  |  |  |  | **intercept** | | | **small shifters** |
| **Accipitriformes** | **intercept**  **(typical species)** | | 55.50 | 1.53 | 52.47 | 58.53 | 36.20 | <0.0001 |  | |  | |
| N_shifter_ = 38  N_typical_ = 120 | **time shifters** | | -7.33 | 3.13 | -13.51 | -1.15 | 36.20 | 0.02 | -0.49 |  | | |
| **Strigiformes** | **intercept**  **(typical species)** | | 29.38 | 0.89 | 27.64 | 31.13 | 33.16 | <0.0001 |  |  | | |
| N_large shifter_ = 28  N_small shifter_ = 43 | **small shifters** | | -10.93 | 1.73 | -14.34 | -7.51 | -6.32 | <0.0001 | -0.51 |  | | |
| N_typical_ = 121 | **large shifters** | | 18.61 | 2.04 | 14.58 | 22.64 | 9.10 | <0.0001 | -0.43 | 0.22 | | |

* Predictor correlations were computed based on the variance-covariance matrix of the corresponding fitted model using the R function cov2cor.

**Table S4.** Phylogenetic informative models of body size differences between time shifters and the typical, non time-shifted species from the orders Accipitriformes and Strigiformes. Shown are estimates with their 95% confidence intervals (CI).

| **model** | **predictor** | **estimate^*^** | | **SE^*^** | **lower**  **CI^‡^** | **upperCI^‡^** | **z^*^** | **P^*^** | **predictor correlations^*^** | | |
| --- | --- | --- | --- | --- | --- | --- | --- | --- | --- | --- | --- |
|  |  |  |  |  |  |  |  |  | **intercept** | | **small shifters** |
| **Accipitriformes** | **intercept**  **(typical species)** | | 67.94 | 10.01 | 63.92 | 72.17 | 6.78 | <0.0001 |  |  | |
| N_shifter_ = 38  N_typical_ = 120 | **time shifters** | | -0.34 | 2.05 | -1.90 | 1.13 | -0.16 | 0.87 | -0.05 | |  |
|  | **lambda**^†^ | | 0.94 | - | 0.94 | 0.98 |  |  |  | |  |
| **Strigiformes** | **intercept**  **(typical species)** | | 27.94 | 6.34 | 27.20 | 28.99 | 4.41 | <0.0001 |  | |  |
| N_large shifter_ = 28  N_small shifter_ = 43 | **small shifters** | | -3.57 | 1.64 | -4.30 | -1.91 | -2.18 | 0.03 | -0.06 | |  |
| N_typical_ = 121 | **large shifters** | | 6.04 | 1.41 | 5.38 | 7.83 | 4.29 | <0.0001 | -0.03 | | 0.05 |
|  | **lambda**^†^ | | 0.86 | - | 0.79 | 0.94 |  |  |  | |  |

* Estimate, SE, z, P values and predictor correlations were taken from the model using the average tree (see Methods). Predictor correlations were computed based on the variance-covariance matrix of the corresponding fitted model using the R function cov2cor.

^†^ Estimated phylogenetic signal (Pagel’s lambda).

**^‡^** 95% confidence intervals (CI) of the estimates were computed by the 2.5% and 97.5% percentiles of the estimates based on 9999 models with 9999 phylogenies.

**Table S5.** Predictors of the number of time-shifted species in the orders Accipitriformes and Strigiformes within zoogeographical realms, including day or night length. Shown are estimates with their upper and lower 95% confidence intervals (CI). The number of time-shifted species and the predictors were standardized.

| **model** | **predictor*** | **estimate** | | **SE** | **lower**  **CI** | **upper**  **CI** | | **z** | **P** | **predictor correlations**^†^ | | | |
| --- | --- | --- | --- | --- | --- | --- | --- | --- | --- | --- | --- | --- | --- |
|  |  |  |  |  |  |  |  |  |  | **intercept** | **total predator** | **day- / night-active predator** | |
| **Accipitriformes** | **intercept** | 0.02 | | 0.16 |  |  | |  |  |  |  |  |  |
| N_assemblage_ = 4036 | **total predator** | 0.40 | | 0.10 | 0.16 | 0.64 | | 3.95 | 0.0002 | 0.48 |  |  |  |
| N_realm_ = 11 | **day-active predator** | 0.20 | | 0.03 | 0.13 | 0.26 | | 7.41 | <0.0001 | 0.01 | -0.26 |  |  |
|  | **day length** | 0.04 | | 0.05 | -0.08 | 0.16 | | 0.77 | 0.82 | 0.07 | 0.02 | -0.04 |  |
|  | **var(realms)** | 0.47 | |  |  |  | |  |  |  |  |  |  |
|  | **var(total predator)** | 0.31 | |  |  |  | |  |  |  |  |  |  |
|  | **var(residual)** | 0.52 | |  |  |  | |  |  |  |  |  |  |
| **Strigiformes** | **intercept** | -0.19 | | 0.19 |  |  | |  |  |  |  |  |  |
| N_assemblage_ = 4036 | **total predator** | -0.15 | | 0.12 | -0.43 | 0.13 | | -1.30 | 0.48 | 0.86 |  |  |  |
| N_realm_ = 11 | **night-active predator** | 0.75 | | 0.01 | 0.72 | 0.79 | | 53.50 | <0.0001 | 0.00 | -0.12 |  |  |
|  | **night length** | -0.10 | | 0.05 | -0.22 | 0.02 | | -2.00 | 0.13 | -0.07 | -0.02 | -0.02 |  |
|  | **var(realms)** | 0.61 | |  |  |  | |  |  |  |  |  |  |
|  | **var(total predator)** | 0.38 | |  |  |  | |  |  |  |  |  |  |
|  | **var(residual)** | 0.44 | |  |  |  | |  |  |  |  |  |  |
| **Small Strigiformes^‡^** | **intercept** | -0.13 | 0.27 | |  |  |  | |  |  |  |  |  |
| N_assemblage_ = 3447 | **total predator** | -0.02 | 0.11 | | -0.29 | 0.25 | -0.19 | | 1.00 | 0.79 |  |  |  |
| N_realm_ = 10 | **night-active predator** | 0.62 | 0.02 | | 0.56 | 0.68 | 24.96 | | <0.0001 | -0.00 | -0.22 |  |  |
|  | **night length** | 0.27 | 0.08 | | 0.07 | 0.47 | 3.16 | | 0.004 | -0.07 | -0.02 | 0.01 |  |
|  | **var(realms)** | 0.82 |  | |  |  |  | |  |  |  |  |  |
|  | **var(total predator)** | 0.33 |  | |  |  |  | |  |  |  |  |  |
|  | **var(residual)** | 0.77 |  | |  |  |  | |  |  |  |  |  |
| **Large Strigiformes^‡^** | **intercept** | -0.17 | 0.17 | |  |  |  | |  |  |  |  |  |
| N_assemblage_ = 4020 | **total predator** | -0.21 | 0.12 | | -0.50 | 0.07 | -1.76 | | 0.21 | 0.87 |  |  |  |
| N_realm_ = 11 | **night-active predator** | 0.69 | 0.01 | | 0.66 | 0.72 | 57.56 | | <0.0001 | 0.00 | -0.10 |  |  |
|  | **night length** | -0.21 | 0.05 | | -0.32 | -0.09 | -4.32 | | <0.0001 | -0.07 | -0.02 | 0.00 |  |
|  | **var(realms)** | 0.55 |  | |  |  |  | |  |  |  |  |  |
|  | **var(total predator)** | 0.39 |  | |  |  |  | |  |  |  |  |  |
|  | **var(residual)** | 0.43 |  | |  |  |  | |  |  |  |  |  |

* Total predator: total number of sympatric avian predator species as an index reflecting exploitation competition pressure; day- / night-active predator: the number of day-/night-active sympatric predator species as an index reflecting interference competition pressure; var(realms), var(total predator) and var(residual) are estimated variance components of the random effects.

^†^ Predictor correlations were computed based on the variance-covariance matrix of the corresponding fitted model using the R function cov2cor.

**^‡^** Small: body size <30 cm; Large: >30 cm (see Methods and figure 2).

**Table S6.** Predictors of the number of time-shifted species in the orders Accipitriformes and Strigiformes within zoogeographical realms, including day or night length. Shown are estimates with their upper and lower 95% confidence intervals (CI). The number of time-shifted species and the predictors were standardized. Strictly migratory species were excluded (see table S5 for all species).

| **model** | **predictor**^*^ | **estimate** | | **SE** | **lower**  **CI** | **upper**  **CI** | | **z** | **P** | **predictor correlations**^†^ | | | |
| --- | --- | --- | --- | --- | --- | --- | --- | --- | --- | --- | --- | --- | --- |
|  |  |  |  |  |  |  |  |  |  | **intercept** | **total predator** | **day- / night-active predator** | |
| **Accipitriformes** | **intercept** | -0.02 | | 0.19 |  |  | |  |  |  |  |  |  |
| N_assemblage_ = 3949 | **total predator** | 0.34 | | 0.10 | 0.09 | 0.59 | | 3.24 | 0.004 | 0.65 |  |  |  |
| N_realm_ = 11 | **day-active predator** | 0.22 | | 0.03 | 0.16 | 0.29 | | 8.67 | <0.0001 | 0.01 | -0.26 |  |  |
|  | **day length** | -0.02 | | 0.05 | -0.14 | 0.10 | | -0.35 | 0.98 | 0.05 | 0.03 | -0.03 |  |
|  | **var(realms)** | 0.59 | |  |  |  | |  |  |  |  |  |  |
|  | **var(total predator)** | 0.32 | |  |  |  | |  |  |  |  |  |  |
|  | **var(residual)** | 0.53 | |  |  |  | |  |  |  |  |  |  |
| **Strigiformes** | **intercept** | -0.15 | | 0.21 |  |  | |  |  |  |  |  |  |
| N_assemblage_ = 3849 | **total predator** | -0.07 | | 0.15 | -0.42 | 0.28 | | -0.51 | 0.94 | 0.90 |  |  |  |
| N_realm_ = 11 | **night-active predator** | 0.75 | | 0.01 | 0.71 | 0.78 | | 49.81 | <0.0001 | -0.01 | -0.11 |  |  |
|  | **night length** | -0.08 | | 0.06 | -0.22 | 0.07 | | -1.27 | 0.50 | -0.05 | -0.01 | -0.00 |  |
|  | **var(realms)** | 0.66 | |  |  |  | |  |  |  |  |  |  |
|  | **var(total predator)** | 0.48 | |  |  |  | |  |  |  |  |  |  |
|  | **var(residual)** | 0.52 | |  |  |  | |  |  |  |  |  |  |
| **Small Strigiformes^‡^** | **intercept** | -0.10 | 0.22 | |  |  |  | |  |  |  |  |  |
| N_assemblage_ = 3054 | **total predator** | -0.06 | 0.11 | | -0.33 | 0.21 | -0.57 | | 0.92 | 0.56 |  |  |  |
| N_realm_ = 10 | **night-active predator** | 0.64 | 0.03 | | 0.57 | 0.71 | 21.83 | | <0.0001 | -0.01 | -0.27 |  |  |
|  | **night length** | -0.08 | 0.10 | | -0.30 | 0.15 | -0.81 | | 0.80 | -0.07 | -0.01 | -0.02 |  |
|  | **var(realms)** | 0.62 |  | |  |  |  | |  |  |  |  |  |
|  | **var(total predator)** | 0.31 |  | |  |  |  | |  |  |  |  |  |
|  | **var(residual)** | 0.81 |  | |  |  |  | |  |  |  |  |  |
| **Large Strigiformes^‡^** | **intercept** | -0.13 | 0.20 | |  |  |  | |  |  |  |  |  |
| N_assemblage_ = 3814 | **total predator** | -0.15 | 0.15 | | -0.51 | 0.21 | -0.99 | | 0.69 | 0.93 |  |  |  |
| N_realm_ = 11 | **night-active predator** | 0.67 | 0.01 | | 0.64 | 0.70 | 53.84 | | <0.0001 | -0.01 | -0.09 |  |  |
|  | **night length** | -0.09 | 0.05 | | -0.22 | 0.03 | -1.81 | | 0.20 | -0.05 | -0.02 | -0.02 |  |
|  | **var(realms)** | 0.64 |  | |  |  |  | |  |  |  |  |  |
|  | **var(total predator)** | 0.49 |  | |  |  |  | |  |  |  |  |  |
|  | **var(residual)** | 0.48 |  | |  |  |  | |  |  |  |  |  |

* Total predator: total number of sympatric avian predator species as an index reflecting exploitation competition pressure; day- / night-active predator: the number of day-/night-active sympatric predator species as an index reflecting interference competition pressure; var(realms), var(total predator) and var(residual) are estimated variance components of the random effects.

^†^ Predictor correlations were computed based on the variance-covariance matrix of the corresponding fitted model using the R function cov2cor.

**^‡^** Small: body size <30 cm; Large: >30 cm (see Methods and figure 2).

**Table S7.** Predictors of the number of time-shifted species in the order Strigiformes within zoogeographical realms, including day or night length. Shown are estimates with their upper and lower 95% confidence intervals (CI). The number of time-shifted species and the predictors were standardized. Here, typical Strigiformes species were defined as those that are explicitly nocturnal, using the same criteria as for typical species in Accipitriformes (see table S5 for models of Accipitriformes and Strigiformes using order-specific classification for typical and time-shifted species).

| **model** | **predictor**^*^ | **estimate** | | **SE** | **lower**  **CI** | **upper**  **CI** | | **z** | **P** | **predictor correlations**^†^ | | | |
| --- | --- | --- | --- | --- | --- | --- | --- | --- | --- | --- | --- | --- | --- |
|  |  |  |  |  |  |  |  |  |  | **intercept** | **total predator** | **day- / night-active predator** | |
| **Strigiformes** | **intercept** | -0.07 | | 0.15 |  |  | |  |  |  |  |  |  |
| N_assemblage_ = 4036 | **total predator** | -0.12 | | 0.11 | -0.39 | 0.14 | | -1.10 | 0.61 | 0.89 |  |  |  |
| N_realm_ = 11 | **night-active predator** | 0.86 | | 0.01 | 0.83 | 0.89 | | 68.98 | <0.0001 | 0.00 | -0.11 |  |  |
|  | **night length** | -0.10 | | 0.04 | -0.20 | 0.00 | | -2.31 | 0.06 | -0.07 | -0.02 | -0.02 |  |
|  | **var(realms)** | 0.47 | |  |  |  | |  |  |  |  |  |  |
|  | **var(total predator)** | 0.36 | |  |  |  | |  |  |  |  |  |  |
|  | **var(residual)** | 0.39 | |  |  |  | |  |  |  |  |  |  |
| **Small Strigiformes^‡^** | **intercept** | -0.07 | | 0.23 |  |  | |  |  |  |  |  |  |
| N_assemblage_ = 3447 | **total predator** | -0.01 | | 0.11 | -0.26 | 0.24 | | -0.07 | 1.00 | 0.85 |  |  |  |
| N_realm_ = 10 | **night-active predator** | 0.61 | | 0.02 | 0.56 | 0.66 | | 27.21 | <0.0001 | -0.00 | -0.22 |  |  |
|  | **night length** | 0.20 | | 0.08 | 0.01 | 0.40 | | 2.49 | 0.04 | -0.05 | -0.02 | 0.01 |  |
|  | **var(realms)** | 0.68 | |  |  |  | |  |  |  |  |  |  |
|  | **var(total predator)** | 0.31 | |  |  |  | |  |  |  |  |  |  |
|  | **var(residual)** | 0.77 | |  |  |  | |  |  |  |  |  |  |
| **Large Strigiformes^‡^** | **intercept** | -0.06 | 0.12 | |  |  |  | |  |  |  |  |  |
| N_assemblage_ = 4020 | **total predator** | -0.19 | 0.11 | | -0.45 | 0.07 | -1.76 | | 0.22 | 0.87 |  |  |  |
| N_realm_ = 11 | **night-active predator** | 0.85 | 0.01 | | 0.82 | 0.88 | 72.54 | | <0.0001 | 0.00 | -0.10 |  |  |
|  | **night length** | -0.20 | 0.04 | | -0.30 | -0.11 | -5.24 | | <0.0001 | -0.08 | -0.02 | 0.01 |  |
|  | **var(realms)** | 0.38 |  | |  |  |  | |  |  |  |  |  |
|  | **var(total predator)** | 0.35 |  | |  |  |  | |  |  |  |  |  |
|  | **var(residual)** | 0.38 |  | |  |  |  | |  |  |  |  |  |

* Total predator: total number of sympatric avian predator species as an index reflecting exploitation competition pressure; day- / night-active predator: the number of day-/night-active sympatric predator species as an index reflecting interference competition pressure; var(realms), var(total predator) and var(residual) are estimated variance components of the random effects.

^†^ Predictor correlations were computed based on the variance-covariance matrix of the corresponding fitted model using the R function cov2cor.

**^‡^** Small: body size <30 cm; Large: >30 cm (see Methods and figure 2).

**Table S8.** Glossary.

| **Accipitriformes** | Hawk and eagle species. Typically diurnal. Some species can be crepuscular. |
| --- | --- |
| **Assemblage** | A group of species that occupies a common geographical region as the unit of analysis. |
| **Avian predators** | Including Accipitriformes, Falconiformes and Strigiformes species. Bird species that kill for food, whereby smaller predator species can be potential prey for larger species. |
| **Crespuscular** | Active during twilight. |
| **Day length** | The period from sunrise to sunset. |
| **Exploitation competition** | Indirect competition among sympatric species by resource depletion without direct contact. |
| **Exploitation competition index** | The number of all sympatric avian predator species, independent of their timing of activity. |
| **Falconiformes** | Falcon species. Typically diurnal or crepuscular. |
| **Interference competition** | Direct competition through defense of resources and imposing harm on competitors. |
| **Interference competition index** | The number of sympatric avian predator species that are active during the day for time-shifted Accipitriformes and during the night for Strigiformes. For large-bodied time-shifted Strigiformes, the index is based only on large sympatric species (body size >30 cm). |
| **Night length** | The period from dusk to dawn. |
| **Species richness** | The number of species. |
| **Strigiformes** | Owl species. Typically nocturnal. Some species are crepuscular or diurnal or cathemeral (i.e. active throughout day and night). |
| **Time-shifters/time-shifted species** | Species active outside the time period typical for that group. |
| **Time-shifted Accipitriformes** | Non-strictly diurnal Accipitriformes species, either 1) active during twilight, or 2) occasionally active at night. |
| **Time-shifted Strigiformes** | Non-strictly nocturnal Strigiformes species, either 1) truly day-active, 2) occasionally active during the day or 3) exclusively crepuscular. |
| **Typical Accipitriformes** | Strictly diurnal Accipitriformes species that are exclusively active during the day. |
| **Typical Strigiformes** | Strictly nocturnal Strigiformes species that are either 1) active exclusively during the night or 2) active during the night and during twilight. |
